# Molecular surveillance on *Streptococcus pneumoniae* carriage in non-elderly adults; little evidence for pneumococcal circulation independent from the reservoir in children

**DOI:** 10.1038/srep34888

**Published:** 2016-10-07

**Authors:** Anne L. Wyllie, Lidewij W. Rümke, Kayleigh Arp, Astrid A. T. M. Bosch, Jacob P. Bruin, Nynke Y. Rots, Alienke J. Wijmenga-Monsuur, Elisabeth A. M. Sanders, Krzysztof Trzciński

**Affiliations:** 1 Paediatric Immunology and Infectious Diseases, Wilhelmina Children’s Hospital, University Medical Center Utrecht, Utrecht, The Netherlands; 2 Spaarne Gasthuis Academy, Hoofddorp and Haarlem, The Netherlands; 3 Regional Laboratory of Public Health, Haarlem, The Netherlands; 4 Centre for Infectious Disease Control, National Institute for Public Health and the Environment (RIVM), Bilthoven, The Netherlands

## Abstract

Carriage of *Streptococcus pneumoniae* in adults is rarely detected by the gold standard culture method. With molecular tests of high sensitivity now available, we analysed upper respiratory tract samples collected during autumn/winter 2012/2013 from parents of PCV7-vaccinated infants and from childless adults, directly comparing culture and qPCR-based *S. pneumoniae* detection. As compared to the gold standard of testing nasopharyngeal swabs, qPCR-based analysis of oral samples significantly improved detection of pneumococcal carriage (5% versus 20%, *p* < 0.0001) with higher carriage rates in parents compared to childless adults (34% versus 7%; *p* < 0.001). Molecular methods also increased the number of serotype-carriage events detected with higher carriage frequencies of serotypes 3 and 7A/F and lower of serotypes 6C/D and 15A/B/C in parents compared to their infant children. We provide evidence that culture-based methods severely underestimate adult carriage rates and for the superiority of testing oral samples over nasopharyngeal swabs. The substantial circulation of pneumococci in parents is however, not representative for the entire adult population. While age-associated differences in serotype carriage suggests reservoirs outside infants as potential sources of vaccine-serotypes contributing to weakening of vaccine herd effects, we find no evidence for reservoirs in adults contributing to serotype replacement in carriage.

Commensal bacterium *Streptococcus pneumoniae* is a frequent coloniser of the human upper respiratory tract^1^. Colonisation may progress to pneumococcal disease, usually manifested (in order of rise in severity) by otitis media, pneumonia, bacteraemia and sepsis, with or without meningitis. Incidence of pneumococcal disease is highest in the extremities of life - in infancy and among the elderly^2^. The main pneumococcal virulence factor is its polysaccharide capsule^3^. While the capsule provides a target for vaccination against *S. pneumoniae*, commercially available vaccines cover only a limited number of the 90+ known capsular types (serotypes)^4^. The purified polysaccharide vaccine (Pneumovax^®^, PPSV23) has the broadest coverage, targeting the 23 serotypes which at the time of PPSV23-introduction in the 1980s, were collectively responsible for 85–90% cases of invasive pneumococcal disease (IPD)^5^. While PPSV23 demonstrates efficacy against IPD in adults^6^, vaccine-effects in infants are poor^7^. Conjugated polysaccharide vaccines (PCVs) however, are proving efficacious in all age groups^8^,^9^ but are of lower serotype coverage. The first PCV targeting seven serotypes was introduced in 2000 (Prevenar^®^, PCV7). The present PCVs, both licensed in 2009, target ten (Synflorix^®^, PCV10) and thirteen (Prevenar-13^®^, PCV13) serotypes. Primarily used to immunise infants, PCVs are also advocated for other individuals at risk of pneumonia and IPD, including aged adults and patients with certain immunodeficiencies^7^.

PCV-vaccination of infants resulted not only in direct protection against childhood disease caused by strains of vaccine serotypes (VT) but also in herd protection against VTs across the entire population^10^,^11^,^12^,^13^. Herd protection can be explained by the reduction in VT carriage and transmission, pointing at young children as the main reservoir of VT strains^14^,^15^. However, reduced circulation of VTs has allowed for the emergence of non-vaccine serotypes (NVT), with near complete replacement of VTs in carriage in vaccinated children and to a lesser extent in disease across the whole population^12^,^13^,^14^,^16^,^17^,^18^. The current understanding of the direct and herd effects of vaccination is based mostly on data on carriage in very young children^19^,^20^ and data on IPD which is dominated by cases in adults^10^,^11^,^12^,^18^. The focus on young children in surveillances on carriage is justified by high colonisation rates of over 50%^15^,^21^ compared to several-fold lower carriage prevalence in other age groups^22^,^23^,^24^,^25^. With few carriers detected, surveillances in adults are considered to be intrinsically underpowered, thus uninformative.

The current gold standard for the detection of pneumococcal carriage is recovery of live pneumococci from cultures of nasopharyngeal swabs and serotyping of cultured strains^26^. Updated recommendations state that when possible, oropharyngeal swabs should be also cultured from adults^27^. However, there is growing evidence for poor sensitivity of the standard culture-based diagnostic methods when applied to detect the presence of *S. pneumoniae* in samples from the upper airways. For example, we recently reported on the superior sensitivity of molecular over culture methods^28^,^29^ and of testing saliva instead of nasopharyngeal or oropharyngeal swabs for detecting carriers among school-aged children^30^ and elderly^31^. In addition, historical records report universally high rates of pneumococcal carriage across all ages when oral samples (oropharyngeal swabs/washes, saliva samples) were tested with very sensitive animal inoculation methods^32^,^33^,^34^. This suggested to us that the contemporary carriage of *S pneumoniae* in healthy adults is underestimated, limiting our understanding of vaccine-effects in the whole population.

After a historical lag of almost 80 years since the last large study in healthy adults^32^,^35^, we investigated pneumococcal carriage using both conventional culture and molecular diagnostic methods applied to nasopharyngeal and oral samples collected from over 600 healthy adults aged 20 to 51 years (non-elderly) in a large cross-sectional study conducted in the Netherlands during the autumn/winter season of 2012/2013. We hypothesised that pneumococcal carriage was underestimated in all adults independent of their parental status or age. We also expected that molecular methods would grant us an insight into serotype carriage in healthy non-elderly adults and help us to understand factors contributing to post-PCV changes in IPD in the Netherlands^12^,^18^, namely herd protection and serotype replacement.

## Methods

### Ethics statement

This study was conducted in accordance with the European Statements for Good Clinical Practice and the declaration of Helsinki of the World Health Medical Association and was approved by METC Noord-Holland (NL40288.094.12)^21^. Written, informed consent was obtained from all study participants.

### Study design

Samples were collected from PCV7-vaccinated infants and unvaccinated adults in a cross-sectional study performed during the autumn/winter season of 2012/2013^21^, 6.5 years post-PCV7 implementation in the Netherlands in 2006 and one year after PCV7 was replaced by PCV10 for all infants born after March 1^st^ 2011. Data from the molecular detection of pneumococcal carriage in children were reported elswehere^29^. In the present study, 330 parents (22 to 51 years old; one per family) of the 24-month-old infants were included as well as 330 healthy adults (20 to 49 years old) with limited contact with children under 6 years of age, defined as less than 8 hours of interaction per week. We refer to the latter participants as childless adults. Detailed descriptions of the parent population and primary study culture results are available elsewhere^21^.

### Sample processing

Samples were collected as previously described^21^,^31^ and transferred within 8 hours to the Regional Laboratory of Public Health in Haarlem. On arrival at the lab, nasopharyngeal and oropharyngeal samples were immediately cultured, as previously reported^28^. Saliva samples were stored at −80˚C until being cultured on SB7-Gent medium plates^31^. Confirmed *S. pneumoniae* strains were serotyped by the Quellung method^21^,^27^,^36^. All remaining bacterial growth was harvested and stored at −80˚C^30^; these samples were considered culture-enriched for *S. pneumoniae*. Samples producing no growth were recorded as negative for *S. pneumoniae*^28^.

### Molecular detection of pneumococci and sample serotype-composition determination

DNA was extracted from 200 μl of culture-enriched samples^30^, eluted as 150 μl template volumes and tested in singleplex quantitative-PCR (qPCR) assays targeting pneumococcal-specific genes *lytA*^37^ and *piaB*^28^,^29^. Samples were considered positive for *S. pneumoniae* when C_*T*_ values for both targeted genes were ≤40^31^,^37^,^38^,^39^. Next, all templates were tested by singleplex-qPCR for *S. pneumoniae* serotype-specific DNA sequences, using a panel of 22 assays targeting individual serotypes or groups of capsular types (serogroups), namely 1, 3, 6A/B/C/D, 7A/F, 8, 10A/B, 12A/B/F, 14, 15A/B/C, 19A, 22A/F, 23F, 35B, 38^40^, 4, 5, 11A/D, 16F, 18B/C, 19F, 23A^41^ and 9A/(N)/V^40^,^41^ (36 serotypes in total). For this, a pooling strategy was implemented. Templates that generated any signal < 45 C_*T*_ for either *lytA* or *piaB* were pooled by five and all templates negative for any signal were pooled by ten. From each pool, 11.5 μl (2.3 μl per template generating *lytA/piaB* signal; 1.15 μl remaining templates) was tested in a total reaction volume of 25 μl. Templates from pools generating any serotype-specific signal < 45 C_*T*_were re-tested individually, using 2.5 μl of template per reaction. Samples were considered positive when the serotype/serogroup-specific signal was ≤40 C_*T*_^31^,^38^,^39^. Serotype-specific qPCR assays which generated signal ≤40 C_*T*_ in any template negative for *lytA* (C_*T*_ = 45), were considered unreliable and excluded from analysis^30^,^31^.

### Statistics

Statistical analyses were conducted using GraphPad Prism v5.0 (GraphPad Software, San Diego, CA, USA). Unless otherwise stated, statistical significance was determined using Fisher’s Exact test and defined as *p* < 0.05.

## Results

Of the 660 adults enrolled, complete sets of upper respiratory tract samples (saliva plus nasopharyngeal and oropharyngeal swabs) were available for analysis from 621 individuals (94%) (parents = 298 of 330, 90%; childless adults = 323 of 330, 98%).

### Pneumococcal carriage detected by culture

Results of *S. pneumoniae* carriage detection using conventional culture for all 621 adults investigated in the current study are reported in Table 1. The fractions of parents identified as colonised based on the conventional culture method in the subsets analysed in this study were not significantly different from the original group (31 of 322, 10%^21^ versus 29 of 298, 10%; *p* = 1.0). Culture-detected carriage rates were significantly higher in parents (n = 29 of 298, 10%) compared to childless adults (n = 5 of 323, 2%; *p* < 0.0001). While more carriers were detected following culture of nasopharyngeal swabs than oropharyngeal swabs for both groups (Table 1), the difference was significant only for parents (*p* < 0.0001) and not for childless adults. Of all sample types, saliva showed the most abundant growth with almost all SB7-Gent culture plates covered by a solid lawn of colonies, rendering isolation of pneumococcal colonies impossible. The least abundant growth was observed for cultures of nasopharyngeal swabs with 68 (23%) of 298 SB7-Gent plates from parents and 175 (54%) of 323 from childless adults negative for any growth. The carriage detection rate was thus reversely correlated with density of (any) bacterial growth on culture plates.

### Pneumococcal carriage rates detected by the molecular method

Since we previously reported that (I) qPCR-based detection of *S. pneumoniae* in nasopharyngeal samples did not significantly increase the number of adult carriers detected compared to conventional culture alone and (II) it did not increase the overall number of adult carriers compared to molecular detection of *S. pneumoniae* in oropharyngeal samples^28^,^31^, and (III) since we recently reported on the superiority of saliva over nasopharyngeal and oropharyngeal samples when tested in elderly with molecular methods^31^, we decided not to investigate nasopharyngeal samples with molecular methods but to target oropharyngeal and saliva samples only. Nevertheless, using qPCR we tested a subset of 80 randomly selected culture-enriched nasopharyngeal samples (parents n = 36, childless adults n = 44) all classified as culture-negative for *S. pneumoniae* despite abundant colony growth. All proved qPCR-negative for *lytA* and *piaB*, giving us confidence that not testing nasopharyngeal samples with molecular methods would not impact study findings.

In line with our findings previously reported for parents^28^, qPCR-based detection of *S. pneumoniae* in culture-enriched oropharyngeal samples alone was superior to culture-detection for nasopharyngeal and oropharyngeal swabs combined (n = 51, 8% versus n = 34, 5% of 621) albeit the difference did not reach statistical significance (Chi-Square, *p* = 0.07) (Table 2). Saliva however, was clearly superior to oropharyngeal swabs when culture-enriched samples were tested with qPCR (n = 107, 17% versus n = 51, 8% of 621; *p* < 0.0001). This superiority was also significant in the individual study groups (parents, saliva n = 89, 30% versus oropharyngeal n = 44, 15% of 298, *p* < 0.0001; childless adults, saliva n = 18, 6% versus oropharyngeal n = 7, 2% of 323, *p* < 0.05). Furthermore, the rate of pneumococcal carriage detected by testing saliva alone was not significantly lower than the overall carriage detected by any method (all adults, n = 107, 17% versus n = 133, 21% of 621, *p* = 0.07; parents, n = 89, 30% versus n = 110, 37% of 298, *p* = 0.082; childless adults, n = 18, 6% versus n = 23, 7% of 323, *p* = 0.519).

Finally, in line with results for culture-based carriage detection, the number of childless adults identified as carriers by qPCR was substantially lower than the number of carriers among parents (n = 21 of 323, 7% versus n = 102 of 298, 34%; *p* < 0.001).

### Serotypes of cultured pneumococcal strains

Thirty-three strains of 15 serotypes and three non-typeable strains (36 isolates in total) were cultured from 33 (5%) of the 621 individuals analysed in this study. In one parent, isolates of the matching serotype were cultured from both the oropharyngeal and nasopharyngeal swabs and were considered to represent a single strain. This reduced the total number of strains cultured in the study to 35 (Table 3). Of note, there was no difference in the prevalence of serotypes detected at the culture step in nasopharyngeal compared to oropharyngeal samples, nor in the carriage of individual serotypes between parents and childless adults. However, with only 29 strains cultured from parents and 6 from childless adults (Table 3) this study was not powered to detect differences in sample type or demographic group when serotype carriage was measured by culture alone.

### Detection of pneumococcal serotypes using the molecular method

Results of molecular serotype detection in adults are listed in Table 3. Our panel of 22 serotype/serogroup-specific qPCR assays targeted 12 (80%) of the 15 serotypes detected by culture. The design of some assays meant they were unable to distinguish individual serotypes within the target serogroup.

### Serotype-specific qPCR assays demonstrating a lack of specificity or sensitivity

As previously observed^30^,^31^, confounding false positive results with amplification curves indistinguishable from those of Quellung-confirmed pneumococcal strains were generated in samples negative for *S. pneumoniae* in assays targeting serotypes/serogroups 4, 5^41^, 12/A/B/F and 35B^40^. We also observed false positivity in the assay published by Azzari *et al*. targeting serogroup 9A/N/V^40^ and in the assay published by Pimenta *et al*.^41^ targeting serotype 23A. For the first time, we observed false positive signals generated in samples negative for pneumococcal-specific sequences, when tested in the serotyping qPCR assays described by Azzari *et al*.^40^ targeting PCV7-VT 14, NVT 22A/F and PCV13-VT 19A. However, unlike in other assays, these false positive signals generated a noticeably different (flattened) amplification curve as compared to those generated by each standard curve and by samples representing genuine serotype-specific signal (concordant curves and/or *lytA* and *piaB* C_*T*_-values matching that of serotype-specific assay) (see Supplementary Fig. S1). Based on this, we could differentiate and exclude from further analysis false positive signals both in samples negative and positive for pneumococcal specific signal.

However, with no sample testing genuinely qPCR-positive for 22A/F (amplification curve matching the standard curve), we tested the 22A/F strains isolated from nasopharyngeal carriage for confirmation of assay sensitivity. As previously observed, the assay targeting serogroup 22A/F again demonstrated a lack of sensitivity with no Quellung-positive strains testing positive by qPCR^29^. We also observed a lack of sensitivity for the assay developed by Pimenta *et al*. targeting serogroup 9A/V^41^. All results from these assays were considered unreliable and excluded. The remaining fifteen assays were classified as reliable and results were analysed. These assays targeted 25 serotypes in total, including 8 (53%) of the 15 serotypes of *S. pneumoniae* strains cultured in the study. Not covered by qPCR were serotypes 9N, 12F, 17F, 22F, 23B, 35B and 35F, representing 12 (36%) of 33 typeable (encapsulated) strains cultured in the study (Table 3). For the subset of serotypes targeted by reliable qPCR assays, application of the molecular method increased the number of oropharyngeal samples (the only sample type collected from all individuals and tested by both conventional and molecular methods, n = 621) positive for a serotype from 4 by culture to 26 by qPCR (*p* < 0.0001). Finally, there was a strong correlation between the serotypes detected by qPCR in oropharyngeal and saliva samples (Spearman’s r = 0.696, *p* = 0.0077), further justifying the testing of saliva with molecular methods (see Supplementary Fig. S2).

### Overall serotype carriage detected by culture and molecular methods

**Figure 1:**
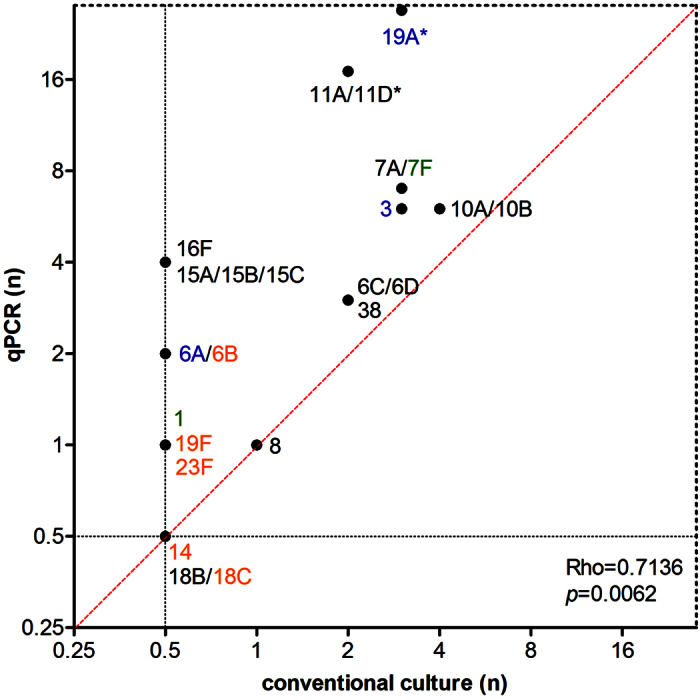
Serotype detection with molecular method (qPCR) versus conventional culture results in all 621 adults investigated in the study. Correlation between the number of individuals positive for serotype as detected by culture, compared to the overall carriers positive for the corresponding serotype by any of the two methods used in the study, for the subset of serotypes targeted by the molecular assay (Spearman’s rho = 0.7136; *p* = 0.0062 after exclusion of serotypes 14 and 18B/C, not detected in carriage). Serotypes not detected by a particular method were assigned a value of 0.5 (half of the lower limit of detection corresponding to a single carrier). Font colour indicates serotypes targeted by PCV7 (orange), PCV10 (green), PCV13 (blue) or NVTs (black). Asterisks depict serotypes which differed significantly (*p* < 0.05) in frequency of carriage between study groups.

Overall, for the subset of serotypes targeted by qPCR, serotype carriage detected by culture correlated to detection by qPCR (Spearman, rho = 0.7136; *p* < 0.01) (Fig. 1). At the level of the individual serotype, application of the molecular method significantly increased detection of PCV13-VT 19A (*p* < 0.0001) and NVT 11A/D (*p* < 0.001), serotypes which were also amongst the most commonly cultured. Of note, with our panel of qPCR assays, we were unable to identify serotypes in samples from 53 (43%) of the 123 carriers detected by the molecular method, including 38 (37%) of 102 carriers among parents and 15 (71%) of 21 carriers among childless adults. This difference between study groups was significant (*p* < 0.01).

### Serotype carriage in adults as compared to 24-month-old infants

**Figure 2:**
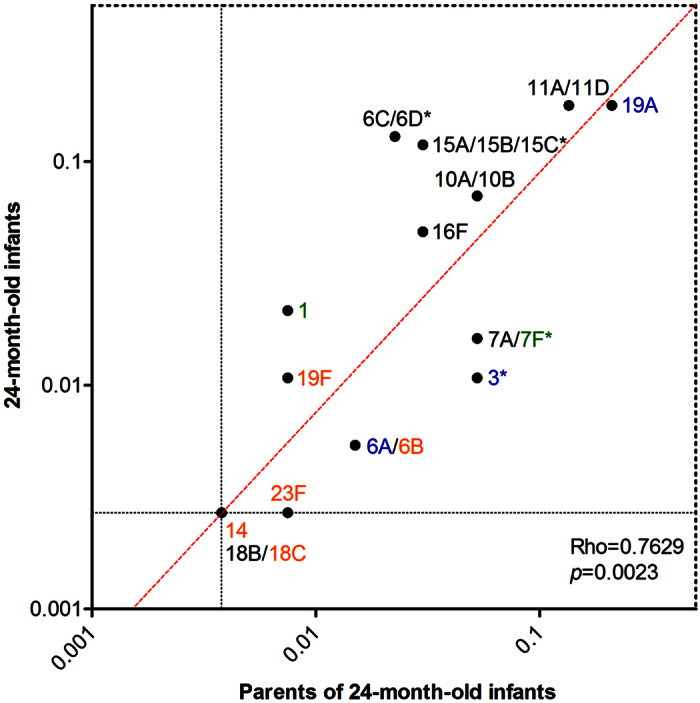
Comparison of overall carriage frequencies of serotypes detected in carriage in parents and their 24-month-old children. Comparison of the overall carriage frequency of serotypes detected by any method in upper respiratory tract samples from 185 carriers of *S. pneumoniae* among 24-month-old infants fully vaccinated with PCV7 and 110 carriers among their unvaccinated parents. Infant samples were obtained from the nasopharynx only. The frequency of carriage was calculated for each serotype by the total number of samples testing positive for particular serotype by either the Quellung or molecular methods, over the total number of pneumococcal carriers detected for each study group. Serotypes absent from carriage in either group were assigned a value of 0.5 x the fraction representing a single carrier. Font colour indicates serotypes targeted by PCV7 (orange), PCV10 (green), PCV13 (blue) or NVTs (black). Asterisks depict serotypes which differed significantly in frequency of carriage between study groups. Since no carriage of serotype 14 or 18B/C was detected in the study they were excluded when the correlation was calculated.

When compared to overall pneumococcal serotype detection in PCV7-vaccinated 24-month-old infants (sampled together with their parents and analysed similarly^29^, see Supplementary Table S1), we observed significantly higher frequencies in carriage of PCV13-VT 3 (*p* = 0.015) and PCV10-associated 7A/F (*p* = 0.04), but lower frequencies of NVTs 6C/D (*p* = 0.003) and 15A/B/C (*p* = 0.02) in carriers among parents compared to carriers among children (Fig. 2). Furthermore, the frequency of PCV13-VTs in carriage among parents was significantly higher than in childless adults (n = 45 of 110, 41% versus n = 2 of 23, 9%; *p* < 0.01). This was mostly due to higher carriage of non-PCV7 serotypes 1, 3, 7A/F and 19A, (n = 41 of 110, 37% versus n = 2 of 23, 9%; *p* < 0.05), and PCV13-unique serotypes 3 and 19A in particular (n = 33 of 110, 30% versus n = 2 of 23, 9%; *p* < 0.05), although none of the differences in individual serotype carriage was significant.

## Discussion

Pneumococcal carriage is an important parameter in studying the effects of PCVs: the direct effects in immunised individuals as well as the indirect effects via any impact on *S. pneumoniae* transmission within the whole population. In the current study, we applied molecular methods to alternative samples from adults in an attempt to improve pneumococcal carriage detection in adults. In line with our previous study in elderly^31^, we provide further evidence for the superiority of molecular surveillance on saliva over nasopharyngeal sampling also in middle-aged adults. However, our results suggest that unlike in parents of very young children^28^ or in the elderly^31^, pneumococcal carriage is virtually absent in childless adults. It confirms that, at least in high-income countries like the Netherlands, children are indeed the major reservoir of pneumococci and the herd effects of infant vaccination are the major force shaping serotype carriage across the whole population.

As recently reported for 24-month-old infants^29^, in the current study qPCR uniformly and proportionally increased the sensitivity of serotype carriage detection compared to culture. This meant that by culture-detection alone, serotypes more frequently carried in the population were also more often under-detected^29^. This included serotypes 3, 7A/F and 11A/D, which in 2012/2013 were isolated from more cases of adult IPD than childhood IPD^8^ and here, also the frequencies in carriage for serotypes 3 and 7A/F were higher in parents than infants^29^. These observations combined suggest that strains of some serotypes may be more associated with adulthood by nature. Age-associated differences in serotype carriage between unvaccinated adults and PCV7-vaccinated infants^29^ could point at reservoirs outside of young children as potential sources of VTs (serotypes 3 and 7A/F in particular) contributing to the weakening of vaccine herd effects. However, it is important to note that in the winter season of 2012/2013, 24-month-old children in the Netherlands had only received PCV7; PCV10 was introduced for newborns born after March 1, 2011. Therefore, any herd effects for the additional PCV10-VTs 1, 5 and 7F were not yet expected.

Although frequencies of serotypes 3 and 7F in carriage were higher in parents than in their infant children, 3 and 7F were virtually absent in the childless adults. Even if infants are not a primary reservoir of these serotypes, presence in children seems to accelerate their transmission in adults. In fact, since no single NVT was more frequent in carriage in childless adults compared to parents, we found no evidence for an independent reservoir in non-elderly adults as a source of NVTs contributing to serotype replacement in carriage.

This supports an assumption that serotypes with high frequencies in carriage in vaccinated-infants, such as serotypes 6C/D and 15A/B/C in this study, may herald an increase in frequency in adult carriage and in disease in near future^20^. However, our panel of serotyping assays was unable to identify the serotype(s) present in a large fraction of adult samples that were qPCR-positive for pneumococci, including the majority of samples from childless adults. Revealing these serotypes with an expansion of reliable assays would be necessary to provide more insight into adult versus child pneumococcal carriage.

While updated WHO recommendations for pneumococcal surveillance in adults state that when possible, oropharyngeal samples should be collected in addition to nasopharyngeal samples, it is still recommended that these samples are processed by the culture-based method^27^. The oral cavity however is more microbially rich than the nasopharynx^42^, making pneumococcal detection by culture difficult for oropharyngeal swabs and virtually impossible for saliva^30^,^31^. As we previously reported^29^, low sensitivity of pneumococcal carriage detection when using the culture-based method is an artefact of both low absolute and low relative abundance of pneumococci in a sample. In the case of adults, the general low absolute abundance of *S. pneumoniae* contributes even further to failure of carriage detection with conventional culture methods. In the current study, only ten of 133 carriers detected were pneumococci-positive from culture of their nasopharyngeal sample alone, inclusion of which, did not significantly affect the overall carriage rate (123, 20% versus 133, 21% of 621, *p* = 0.48). Conversely, 62 carriers were positive for pneumococci solely in saliva, which significantly contributed to the increase in carriage detected from 71 (11%) to 133 (21%) (Chi-Square *p* < 0.0001). These findings highlight the requirement for the inclusion of saliva samples as done so historically in carriage studies in adults^35^.

When transitioning to culture-independent methods for pneumococcal detection and serotyping, strict controls for monitoring assay sensitivity and specificity are imperative. We show this in the current study with false positive signals generated from qPCR assays which we and others have previously classified as reliable for use in molecular-based surveillances on carriage^29^,^30^,^31^,^41^,^43^. It is well established that the oral niche contains a greater prevalence of assay-confounding non-pneumococcal bacteria as compared to the less microbially diverse nasopharynx^42^,^44^. In line with this, qPCR-serotyping assays which previously proved reliable in nasopharyngeal samples^29^ generated false positive signals when applied here to oropharyngeal and saliva samples. This observation highlights the importance of continued scrutiny when applying molecular serotyping methods to complex respiratory samples. Samples negative for pneumococci must always be tested, with results stringently checked, particularly for assays targeting VTs or prevalent NVTs to prevent overrepresentation or misinterpretation of vaccine effects on carriage. Despite the qPCR-based method being reported as one of the most sensitive for serotype detection in polymicrobial samples^45^, this study demonstrates the need for further expansion of reliable assays. Broader serotype coverage is essential for accurate pneumococcal surveillance in this post-vaccination era, particularly when adult pneumococcal vaccination is considered^46^.

In conclusion, contemporary adult pneumococcal carriage rates are largely underestimated when only culture-based methods are applied and more so, when only nasopharyngeal samples are analysed. We demonstrate the need for sampling the oral niche and the use of molecular methods for enhanced pneumococcal detection. Our study raises important considerations for further investigations of pneumococcal reservoirs in adults, which outside of young children represent potential sources of VTs contributing to weakening of the vaccine herd effects. However, we failed to find any substantial evidence for the circulation of *S. pneumoniae* strains in carriage being independent from the reservoir in children, or for the reservoir in non-elderly adults being a source of serotypes contributing to serotype replacement. Nevertheless, our findings support the sampling of parents in surveillance studies as a key measurement of pneumococcal-vaccination herd protection in adults and/or unvaccinated individuals.

## Additional Information

**How to cite this article**: Wyllie, A. L. *et al*. Molecular surveillance on *Streptococcus pneumoniae* carriage in non-elderly adults; little evidence for pneumococcal circulation independent from the reservoir in children. *Sci. Rep*. **6**, 34888; doi: 10.1038/srep34888 (2016).

## Supplementary Material

Supplementary Information

## Figures and Tables

**Table 1:** Detection of *Streptococcus pneumoniae* in upper respiratory tract samples of non-elderly adults by conventional diagnostic culture.

Sample type	Study group		
	Parents (n = 298)	Childless adults (n = 323)	Overall (n = 621)
Nasopharyngeal	26 (9%)	4 (1%)*	30 (5%)
Oropharyngeal	4 (1%)^#^	2 (1%)	6 (1%)^#^
Saliva	0^#^	0	0
Overall	**29 (10%)**	5 (2%)*	**34 (5%)**

*p < 0.0001 (Fisher’s Exact) for comparison of results from parent and childless adult study groups.

^#^*p* < 0.0001 (Fisher’s Exact) for comparison between the number of carriers detected by this sample type and overall number of carriers detected by culture, reported in the last row. Only statistically significant differences are marked.

**Table 2:** Detection of *Streptococcus pneumoniae* in upper respiratory tract samples of adults by molecular diagnostic methods.

Sample type	Study group					
	Parents (n = 298)	sensitivity	Childless adults (n = 323)	sensitivity	Overall (n = 621)	sensitivity
Nasopharyngeal	NA	—	NA	—	NA	—
Oropharyngeal	44 (15%)	0.43^##^	7 (2%)*	0.33^#^	51 (8%)	0.41^##^
Saliva	89 (30%)	0.87	18 (6%)*	0.86	107 (17%)	0.87
Total	**102 (34%)**	**1.0**	21 (7%)*	**1.0**	**123 (20%)**	**1.0**

*p < 0.0001 (Fisher’s Exact) for comparison of results from parent and childless adult study groups.

^#^*p* < 0.05, ^##^*p* < 0.0001 (Fisher’s Exact) for comparison between the sensitivity of this sample type as compared to the sensitivity of all molecular method results combined.

NA – not tested.

**Table 3:** Overall number of carriers positive for serotypes as detected by conventional culture and serotype-specific signals detected by molecular method (qPCR) among the 133 adults identified as carriers of *S. pneumoniae* by any method used in the study.

Serotypes/serogroups	Parents (n = 110)			Childless adults (n = 23)			Overall (n = 133)		
	Culture	qPCR	Total^a^	Culture	qPCR	Total^a^	Culture	qPCR	Total^a^
1^*PCV10*^#	0	1	**1**	0	0	**0**	0	1	**1**
3^*PCV13*^#	3	6	**7**^e^	0	0	**0**	3	6	**7**
4^*PCV7*^#	0	NS^b^	**0**	0	NS	**0**	0	NS	**0**
5^*PCV10*^#	0	NS	**0**	0	NS	**0**	0	NS	**0**
6A^*PCV13*^/6B^*PCV7*^#	0	2	**2**	0	0	**0**	0	2	**2**
6C/6D	2/0^c^	3	**3**	0	0	**0**	2/0	3	**3**
7A/7F^*PCV10*^#	0/3	7	**7**	0	0	**0**	0/3	7	**7**
8#	1	0	**1**^e^	0	1	**1**	1	1	**2**
9A/9N#/9V^*PCV7*^#	0	NS	**0**	0/1/0	NS	**1**	0/1/0	NS	**0**
10A#/10B	4/0	5	**6**^e^	0	1	**1**	4/0	6	**7**
11A#/11D	2/0	16	**17**	0	1	**1**	2/0	17	**18**
12A/12B/12F#	0/0/1	NS	**1**	0	NS	**0**	0/0/1	NS	**1**
14^*PCV7*^#	0	0	**0**	0	0	**0**	0	0	**0**
15A/15B#/15C	0	4	**4**	0	0	**0**	0	4	**4**
16F	0	4	**4**	0	0	**0**	0	4	**4**
17F#^d^	1	—	**1**	0	—	**0**	1	—	**1**
18B/18C^*PCV7*^#	0	0	**0**	0	0	**0**	0	0	**0**
19A^*PCV13*^#	3	25	**26**^e^	0	2	**2**	3	27	**28**
19F^*PCV7*^#	0	1	**1**	0	0	**0**	0	1	**1**
21^d^	0	—	**0**	0	—	**0**	0	—	**0**
22A/22F#	0/1	NS	**1**^e^	0/1	NS	**1**^e^	0/2	NS	**2**
23A	0	NS	**0**	0	NS	**0**	0	NS	**0**
23B^d^	3	—	**3**	0	—	**0**	3	—	**3**
23F^*PCV7*^#	0	1	**1**	0	0	**0**	0	1	**1**
31^d^	0	—	**0**	0	—	**0**	0	—	**0**
35B	1	NS	**1**	1	NS	**1**	2	NS	**2**
35F^d^	1	—	**1**	1	—	**1**	2	—	**2**
38	1	2	**2**	1	1	**1**	2	3	**3**
NT^d^	2	—	**2**	1	—	**1**	3	—	**3**
Total	29	77	**92**	6	6	**11**	35	83	**103**

^*PCV7*^serotype targeted by all three pneumococcal conjugate vaccines (PCVs); ^*PCV10*^serotype targeted by PCV10 and PCV13 only; ^*PCV13*^serotype targeted by PCV13 only.

^#^Serotype targeted by PPSV23 (PPSV23 serotypes 2 and 20 not detected by culture nor targeted by molecular assays).

^a^Total number of carriers positive for the particular serotype.

^b^NS, assay considered non-reliable due to lack of specificity.

^c^n/n, serotype-specific conventional culture results for serotypes indistinguishable from the serogroup when targeted by qPCR, numbers correspond to serotypes reported in the first column.

^d^Serotype not targeted by qPCR assays available thus detected only by conventional culture.

^e^Discrepancies in serotype detection by culture and qPCR due to in individuals with *S. pneumoniae* cultured from nasopharyngeal sample yet oropharyngeal and saliva samples negative for *S. pneumoniae* by the molecular method.
