# Supplementary material for: Molecular surveillance on Streptococcus pneumoniae carriage in non-elderly adults; little evidence for pneumococcal circulation independent from the reservoir in children
Source: Sci Rep. 2016 Oct 7;6:34888. doi: 10.1038/srep34888 (PMC5054371; doi:10.1038/srep34888)
Supplement: Supplementary Information [file srep34888-s1.pdf]

Molecular surveillance on *Streptococcus pneumoniae* carriage in non-elderly adults; little evidence for pneumococcal circulation independent from the reservoir in children.

Anne L. Wyllie[1], Lidewij W. Rümke[1], Kayleigh Arp[1], Astrid A.T.M. Bosch[1], Jacob P. Bruin[2], Nynke Y. Rots[3], Alienke J. Wijmenga-Monsuur[3], Elisabeth A.M. Sanders[1,3] and Krzysztof Trzciński[1]*

[1]Paediatric Immunology and Infectious Diseases, Wilhelmina Children's Hospital, University Medical Center Utrecht, Utrecht, the Netherlands; [2]Regional Laboratory of Public Health, Haarlem, the Netherlands; [3]Centre for Immunology of Infectious Diseases and Vaccines, National Institute for Public Health and the Environment (RIVM), Bilthoven, the Netherlands.

Supplementary Table S1. Overall number carriers positive for serotypes detected by conventional culture and serotype-specific signals detected by molecular method (qPCR) among the 185 24-month-old infants identified as carriers of *S. pneumoniae* by any method used in the study[1].

	**24-month-olds (n=185)**[1]		
**Serotypes /serogroups**	Culture	qPCR	**Total**[a]
**1**[*PCV10#*]	3	4	**4**
**3**[*PCV13#*]	1	2	**2**
**4**[*PCV7#*]	0	NS[b]	**0**
**5**[*PCV10#*]	0	NS	**0**
**6A**[*PCV13*]**/6B**[*PCV7#*]	1/0[c]	1	**1**
**6C/6D**	21/0	24	**24**
**7A/7F**[*PCV10#*]	0/3	3	**3**
**8**[#d]	0	-	**0**
**9A/9N**[#]**/9V**[*PCV7#*]	0/1/0	2	**3**
**10A**[#]**/10B**	9	13	**13**
**11A**[#]**/11D**	19	33	**33**
**12A/12B/12F**[#]	0	NS	**0**
**14**[*PCV7#*]	0	0	**0**
**15A/15B**[#]**/15C**	4/8/3	21	**22**
**16F**	6	9	**9**
**17F**[#d]	3	-	**3**
**18B/18C**[*PCV7#*]	0/0	0	**0**
**19A**[*PCV13#*]	23	33	**33**
**19F**[*PCV7#*]	2	2	**2**
**21**[d]	8	-	**8**
**22A/22F**[#]	0/2	NS	**2**
**23A**	9	NS	**9**
**23B**[d]	18	-	**18**
**23F**[*PCV7#*]	0	0	**0**
**31**[d]	1	-	**1**

**35B**[d]	4	**-**	**4**
**35F**[d]	6	**-**	**6**
**38**[d]	3	**-**	**3**
**NT**[d]	2	**-**	**2**
**Other**[d]	6	**-**	**6**
**Total**	166	153	**213**

[PCV7]serotype targeted by all three pneumococcal conjugate vaccines (PCVs); [PCV10]serotype targeted by PCV10 and PCV13 only; [PCV13]serotype targeted by PCV13 only

[#]serotype targeted by PPSV23 (PPSV23 serotypes 2 and 20 not detected by culture nor targeted by molecular assays).

[a]total number of carriers positive for the particular serotype.

[b]NS, assay considered non-reliable due to lack of specificity.

[c]n/n, serotype-specific conventional culture results for serotypes indistinguishable from the serogroup when targeted by qPCR, numbers correspond to serotypes reported in the first column.

[d]serotype not targeted by qPCR assays available thus detected only by conventional culture.

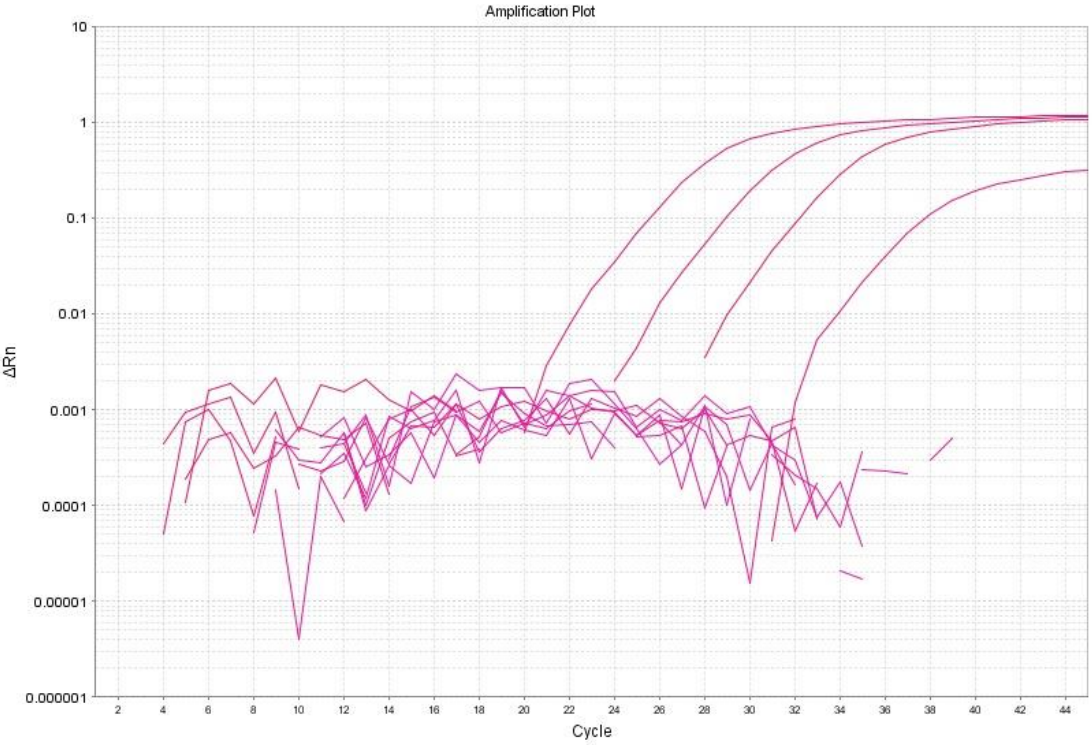
Amplification Plot
10
1
0.1
0.01
0.001
0.0001
0.00001
0.000001
ΔRn
2
4
6
8
10
12
14
16
18
20
22
24
26
28
30
32
34
36
38
40
42
44
Cycle

(a)

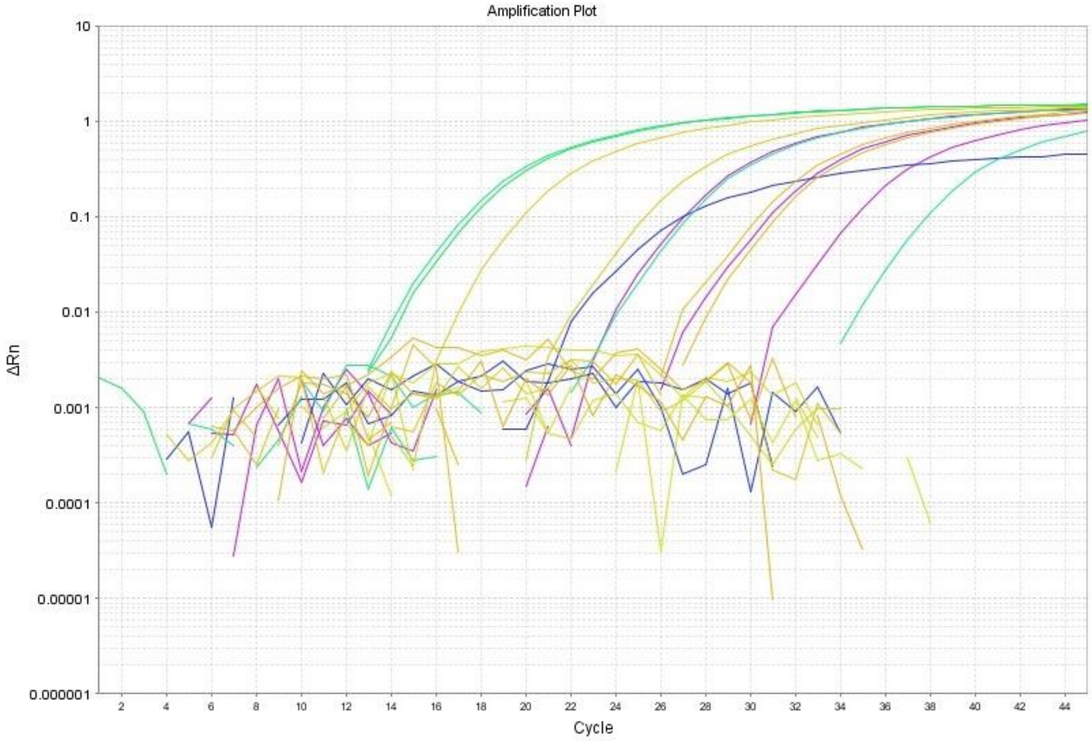
Amplification Plot
10
1
0.1
0.01
0.001
0.0001
0.00001
0.000001
ΔRn
2
4
6
8
10
12
14
16
18
20
22
24
26
28
30
32
34
36
38
40
42
44
Cycle

(b)

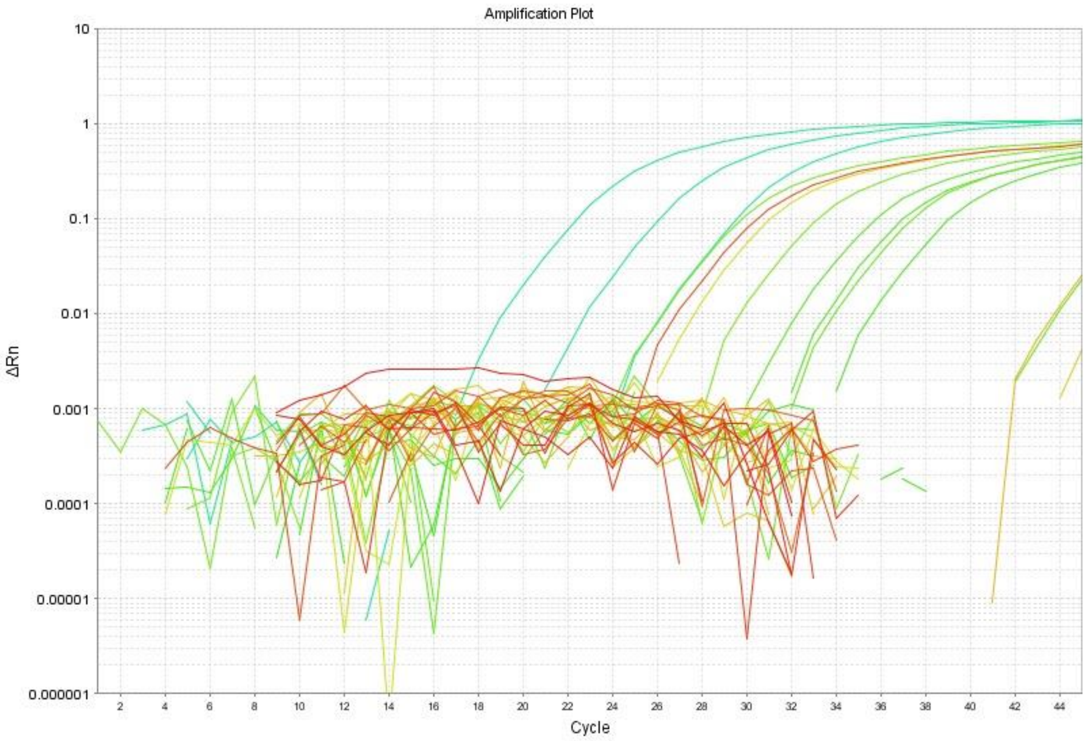

(c)

Supplementary Figure S1. False positive signals producing lower amplification curves in qPCR-serotyping assays when applied to culture-enriched oral samples from adults. Depicted are the qPCR amplification curves generated by assays targeting serotypes (a) 14, (b) 19A and (c) 22A/F when applied to culture-enriched samples from adults included in the current study. Amplification curves with noticeably lower plateaus as compared to the standard curve are visible, all of which were generated from samples completely negative (C_T=45) for pneumococcal specific-specific genes *lytA* and *piaB.* These curves therefore represent culture-enriched samples false positive for serotype-specific gene target. (14, standard curve=higher amplification lines, genuine signal=none, false positive signal=lower amplification line; 19A, standard curve=purple lines, genuine signal=green and yellow lines, false positive signal=blue line; 22A/F, standard curve=green/blue lines, genuine signal=none, false positive signal=red, yellow and light green lines).

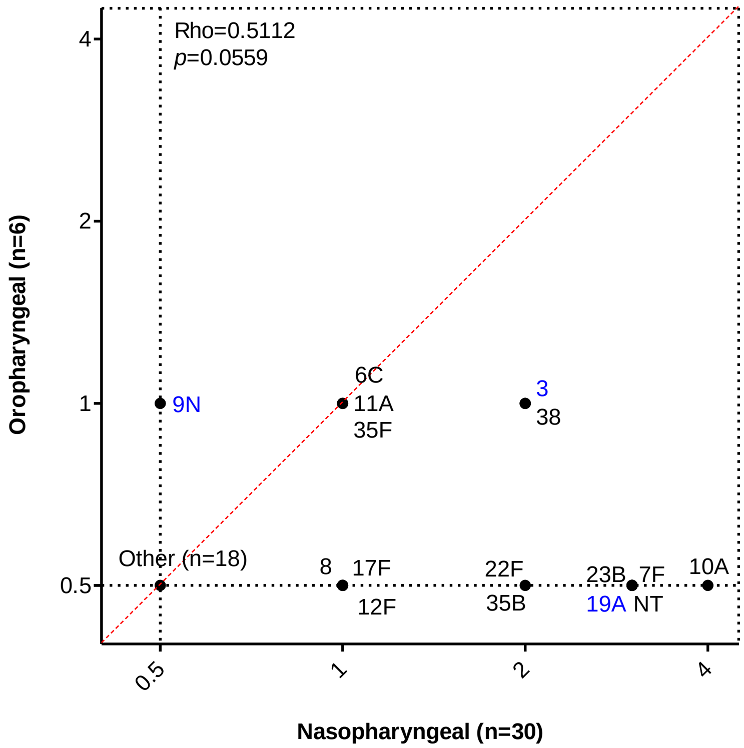

(a)

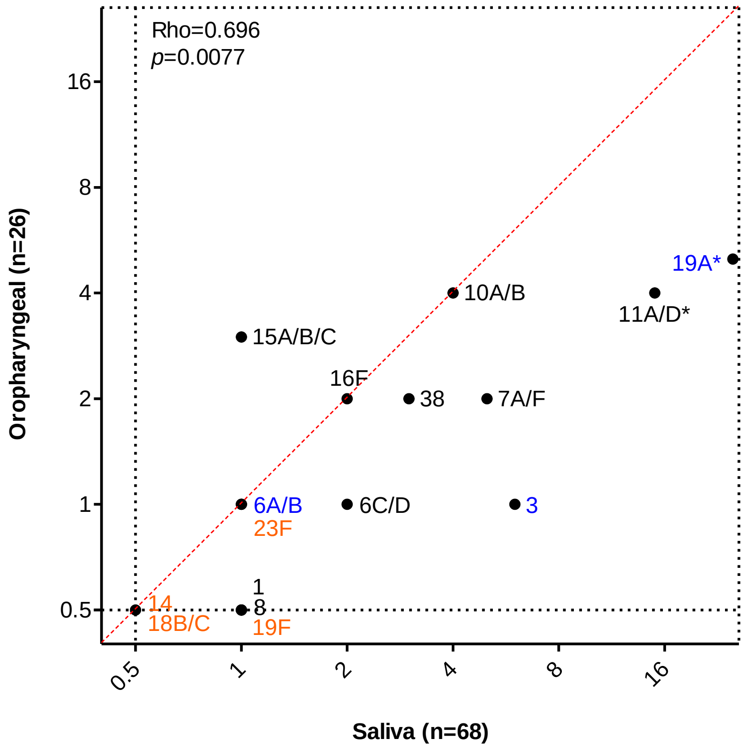

(b)

Supplementary Figure S2. Improved detection of *Streptococcus pneumoniae* serotypes in upper respiratory tract samples when the molecular method is applied as compared to conventional culture. Correlation between the detection of *S. pneumoniae* serotypes (subset

targeted by qPCR assays) in (a) nasopharyngeal and oropharyngeal swabs from all 621 adults included in the study when tested by conventional culture only and the concordance between serotype detection in (b) oropharyngeal and saliva samples when processed by both culture and molecular methods was also tested. Serotypes absent from one sample type, which were present in the other, were assigned a value of half the level of detection (0.5) to allow for a correlation to be made. Font colour indicates serotypes targeted by PCV7 (orange), PCV10 (green), PCV13 (blue) or NVTs (black).

REFERENCES

1. Wyllie, A. L. *et al.* Molecular surveillance of nasopharyngeal carriage of Streptococcus pneumoniae in children vaccinated with conjugated polysaccharide pneumococcal vaccines. *Sci. Rep.* **6,** 23809 (2016).
